# Restricted Science

**DOI:** 10.3389/fpubh.2014.00158

**Published:** 2014-09-24

**Authors:** Sonia Ben Ouagrham-Gormley, Shannon R. Fye

**Affiliations:** ^1^Biodefense Program, School of Policy, Government and International Affairs, George Mason University, Fairfax, VA, USA

**Keywords:** H5N1, NSABB, regulating dual-use research, Venter Institute, *M. mycoides*, bioweapon

## Introduction

In 2004, the National Science Advisory Board for Biosecurity (NSABB) was created as an independent federal advisory body. Its role was to advise the U.S. government on strategies to prevent the misuse of dual-use research. Since its inception, the NSABB has ruled on two cases: the 1918 flu-virus synthesis conducted by government scientists in 2005 and the H5N1 experiment conducted in 2011 by two separate university teams in the Netherlands and the United States. While in the first case, without much public debate, the NSABB quickly decided to support publication of the experiment’s findings, in the second case, it initially requested a halt on publication and the removal of methodological details from the proposed articles for fear that they could be used by malevolent actors to create a pandemic among humans. The decision was reversed 6 months later, but it sparked a worldwide firestorm, engaging the scientific and security communities in a heated debate about whether the dissemination of scientific data should be regulated, and what types of research should be conducted. Yet, the key question that triggered the overall controversy remains largely ignored: under what conditions could the H5N1 experiment be reproduced, if at all, by malevolent actors using only published data?

The lack of attention to the issue of reproducibility stems from a widespread belief that science is inherently reproducible and published data are the primary tool allowing such replication. Empirical evidence suggests otherwise. Analysis of recent dual-use research projects and past bioweapons programs shows that reproducibility of past work faces stiff challenges, especially when using written protocols alone. Translating a scientific idea into a product that functions reliably is a challenge that is routinely encountered in the pharmaceutical industry, as well as in past bioweapons programs. In this article, we start by emphasizing the challenges associated with reproducing scientific experiments and their application to specific purposes based on empirical research conducted by the authors. We then suggest criteria to weigh security risks against the health benefits of dual-use research for the purpose of producing more accurate threat assessments, without imposing unnecessary restrictions on the diffusion of knowledge.

## Sources of Reproducibility Challenges in Science

While the H5N1 controversy was raging, the National Institutes of Health (NIH) revealed that much of its past funded research could not be reproduced. In 2012, for example, the drug company Amgen reported that it failed to reproduce 89% of the findings from 53 major cancer-related papers (1). The previous year, the pharmaceutical company Bayer in Germany indicated that it could not validate the results of two-thirds of its own preclinical studies (1). Interestingly, no connections were made between these revelations and the H5N1 experiment, also funded by the NIH.

Empirical research shows that some experiments are extremely difficult to replicate, due to the contingencies associated with experimental work and the nature of knowledge. First, replication of past work using published documents is problematic because scientific articles rarely provide a detailed account of all stages of an experiment and their associated contingencies. The methods section of scientific papers is usually brief and provides only an overview of the experimental methods to show that a concept has been implemented; it is not intended to be a step-by-step protocol (2). Second, scientific articles rarely delve into the problems that researchers encountered during the experiment nor do they explain how long it took to resolve such problems. For example, the article describing the 2010 creation of a self-replicating *Mycoplasma mycoides* cell by researchers at the J. Craig Venter Institute (JCVI) includes a two-sentence statement indicating that the team faced challenges with transplantation, which were eventually overcome (3). However, interviews with JCVI scientists reveal that transplantation attempts routinely failed for 2 years, leading the scientist responsible for transplantation to consider abandoning the project. As her supervisor explains:

After two years of just seven days a week [of continuous work], she came into my office saying she wanted to work on a new project; she couldn’t do this anymore …. We tried lots and lots of different approaches. And we had suspicions of something we thought might work … but these were hard experiments to do with a lot of reagent prep for every experiment …. Everything you could possibly think of that might allow you to move a really big piece of DNA into a cell [we tried] (4).

Publications often play down the long and painstaking process of systematic problem solving that is often required to resolve difficulties involved in experimental work, leaving the false impression that problems can be readily overcome.

Experimental work also sometimes requires the development of new techniques and protocols that cannot easily be used for other purposes or by other individuals. In the *M. mycoides* transplantation case, a new protocol had to be designed for the experiment, and was published in 2007. Yet, 6 years later, the researchers were not able to use this protocol for work with another organism (4). Additionally, the researchers worked with large pieces of DNA that break easily during pipetting, introducing an additional hurdle to replicating the experiment. To prevent damage, the team emphasized the importance of pipetting “gently” and using pipette tips with wide openings through which large pieces of DNA could pass unobstructed (5). Although pipetting is a common technique, not all scientists were able to pipette the *M. mycoides* DNA gently enough to keep it intact. As one researcher explains:

Our genome transplanters are really good at this [keeping supercoiled DNA intact] … I sat in the same hood … with Carole [Lartigue – the expert] and we used the same reagents … the only thing different was each of us had our own pipettes and plates, and I did a transplant in parallel with her … she got … 2,000 colonies [successful transplants] and I got 20. I thought I was doing exactly what she was doing in pipetting slowly. [But] doing these tricks is still very much a magic hand sort of thing (4).

This highlights a problem well known among practicing scientists but generally ignored in evaluations of the potential reproducibility of dual-use experiments: the importance of expertise acquired through years of practice in the laboratory. Much of this expertise involves tacit skills not easily translated into words, such as the muscle memory that allows a researcher to know what constitutes “gentle” pipetting, or acquired and replicated by others, even when a technique is demonstrated in person or an experiment is done in cooperation with the technique’s designer (6–8). Moreover, laboratory disciplines and routines often contribute to the development of laboratory-specific skills that cannot be standardized or transferred to a new location. The University of New York-Stony Brook virologists who synthesized poliovirus in 2002 emphasized the importance of maintaining “sameness” in their laboratory routines, materials, and technicians to ensure successful results. Tellingly, a post-doctoral fellow who spent 6 years in the New York laboratory could not replicate his work in his home laboratory in Belgium (9).

Thus, the tacit, personal, and local nature of knowledge constitutes a strong barrier to reproducibility. Because know-how does not easily translate into words, its importance for experimental success is frequently ignored in threat assessments.

## Application to Nefarious Objectives

The NSABB’s initial decision to edit the H5N1-related article before its publication was followed by the Dutch government’s decision to impose export-control restrictions on the Dutch team’s article. Dutch authorities claimed that the research fell under European Council Regulation EC 428/2009, which attempts to prevent the spread of nuclear, chemical, and biological weapons by requiring an export license before publication (10). These moves are based on the assumption that innovations achieved in the laboratory can be easily fashioned into a harmful agent or a bioweapon. Yet, past bioweapons work shows that transforming a scientific concept developed in the laboratory into a product that has a specific, applied purpose, and functions reliably and effectively can take several decades and require a variety of expertise. Specifically, the passage from laboratory concept to specific application faces the challenge of scaling-up fragile microorganisms for large-scale production and developing a delivery mechanism that will protect the agents from environmental degradation when released as a weapon. For example, within the Soviet bioweapons program, the development of an antibiotic-resistant strain of the bacterium that causes plague took 20 years to achieve and involved teams at three institutes. Scaling-up anthrax and smallpox weapons took Soviet researchers about 5 years to achieve and required the involvement of large teams of scientists, including the designers of the original strains. And within the U.S. bioweapons program, scientists discovered that the botulinum toxin weapon they had produced eventually lost some of its toxicity upon aerosol release. These examples demonstrate that laboratory successes do not necessarily lead to successful application to a specific purpose. Instead, specialized skills honed over years of practice in production and weaponization work are critical to success (11).

## New Analytical Framework

Seen against this background, fears that the H5N1-related articles might support replication by malevolent actors seem exaggerated. They ignore the fact that science is a cumulative process where knowledge is acquired and built through many years of personal and collective experimentation. Therefore, it is neither easily acquired nor easily transferred, and even less so by means of published articles. More importantly, these fears also indicate that the NSABB’s initial decision to edit the H5N1 article before its publication was not rooted in a risk/benefit analysis that considered the determinants of success in scientific work. Indeed, even though the Board interviewed the lead authors and a variety of influenza experts, it did not interview the scientists and technicians who actually conducted the laboratory work (12). In fact, important details about the experiment’s difficulty were revealed after the Board issued its recommendation, and only as a result of the controversy, not as a result of the Board’s inquiry.

Therefore, any future review of dual-use research should be based on a careful analysis of the tacit, personal, and laboratory-specific skills required to perform scientific experiments. This implies that NSABB reviewers conduct face-to-face interviews with the scientists and technicians who executed the laboratory work to identify the hidden contingencies associated with key stages of an experiment, including the development of laboratory- or agent-specific techniques or protocols that may not transfer easily to a new location. A laboratory visit may also reveal hidden laboratory idiosyncrasies that contribute to experimental success and may prevent replication elsewhere. In order to improve the NSABB’s ability to assess the ease of replication by terrorists or states, its reviewers should also include an expert who has hands-on experience working with the microorganism under consideration. In the H5N1 case, NSABB members had access to outside influenza experts, but their lack of experience working with the influenza virus itself, notwithstanding their expertise in other areas, did not allow some of them to appreciate the importance of experimental details that could have impacted the ultimate threat assessment^1^. Without a major change in the NSABB’s approach, future restrictions might result in two equally negative consequences. First, suspicions among foreign entities that restrictions on scientific work are hiding U.S. government bioweapons work might increase. Second, scientists may avoid U.S.-funded research for fear that the government might block their work from being published. To wit, the Dutch scientist who conducted the H5N1 research temporarily blocked by the NSABB recently published a follow-up study in the journal *Cell*. In the Section “Acknowledgment,” he stipulated that the work was not funded by the NIH (13).

## Conflict of Interest Statement

The authors declare that the research was conducted in the absence of any commercial or financial relationships that could be construed as a potential conflict of interest.

## Footnotes

^1^A list of NSABB members can be found on the National Institute of Health website. A review of their biographies indicate that their respective expertise lies in areas different from the flu: href="http://osp.od.nih.gov/sites/default/files/resources/NSABB%20Voting%20Members%20Roster%20_June%202014_WEB_0.pdf
